# Crack Detection in Metallic Structures Using Planar Monopole Antenna

**DOI:** 10.3390/s25237150

**Published:** 2025-11-23

**Authors:** Nandana Radhakrishnan, Massimo Donelli, Sreedevi K. Menon

**Affiliations:** 1 Department of Physics, Amrita Vishwa Vidyapeetham, Amritapuri 690525, India; am.ps.r4phy25005@am.students.amrita.edu; 2 Department of Civil Environmental and Mechanical Engineering, University of Trento, 38123 Trento, Italy; massimo.donelli@unitn.it; 3 Department of Electronics and Communication Engineering, Amrita Vishwa Vidyapeetham, Amritapuri 690525, India; 4 Centre for Flexible Electronics and Advanced Materials, Amrita Vishwa Vidyapeetham, Amritapuri 690525, India

**Keywords:** antenna sensor, crack detection, monopole antenna, Structural Health Monitoring (SHM), Sustainable Development Goals 9 (SDG 9), SDG 11

## Abstract

**Highlights:**

**What are the main findings?**

- A monopole antenna is established as a crack sensor for Structural Health Monitoring (SHM) applications.
- Distinguishable sensitivities of −41.2 MHz/mm and −30.2 MHz/mm are observed along the crack propagation directions (X and Y) on the ground plane.

**What are the implications of the main findings?**

- The proposed sensor can be attached to planar metallic surfaces to monitor the health condition of the structure.
- Among the other Radio Frequency (RF) sensors, the proposed monopole antenna sensor is unique due to its higher sensitivity, compactness and ease of fabrication.

**Abstract:**

In this paper, a monopole antenna is devised to detect the presence of a crack for Structural Health Monitoring (SHM) application. The performance parameters of the antenna sensor are numerically evaluated using Finite Element Analysis (FEA) and experimentally validated. A crack is introduced as a perturbation in the ground plane of the monopole antenna, resembling the metallic part of the structure that is monitored. The linear resonance frequency shifts in the monopole antenna validate the presence and propagation of a crack in the structure under observation. Different orientations and dimensions of the crack are analyzed to study the sensitivity of the monopole antenna as a sensor for crack detection with a higher sensitivity of −41.2 MHz/mm. The influence of the crack on the gain and radiation patterns is also addressed to evaluate the performance degradation. The simulation and experiment results ensure the ability of the proposed antenna sensor to detect minute structural defects in metallic structures.

## 1. Introduction

Monuments, bridges, and a countless number of other man-made structures require proper inspection and management to have a long life. Failure to do so will result in the formation of structural defects that can grow to become a serious threat to the stability of the structure. Structural Health Monitoring (SHM) is a measure that will solve this difficulty. Real-time analysis of structural damage is critical for sensing surface discontinuities that are manifested over time [1]. These discontinuities can be deformations or cracks that are developed due to large service time span, environmental stresses, corrosion of materials and heavy loads on the structure [2,3]. The defect on the surface indicates serious damage to the underlying layers of the structure. Proper monitoring of the presence and propagation of cracks is vital for maintaining structural integrity and proper maintenance of the property [4,5,6]. Structural response measurements help in the detection of cracks and provide details on the defect’s characteristics, such as its initiation, development, propagation, size, and location [7].

Crack sensing relies mostly on visual inspection, but this manual method is time-consuming and is unable to provide early warning in physically large structures [8]. Subsequently, different advancements in methods for detecting cracks like fiber-optic sensors [9], piezoelectric-based sensors [10,11], and piezoelectric transducers [12] have come into the picture, which are more reliable for monitoring the structures. Passive wireless sensors, such as antennas, can provide an easy translation of defects, which can be made to sense wirelessly, thus reducing the complexities [13]. Antenna-based sensors detect variations in electromagnetic parameters to detect deformation. The antenna sensors limit the dependence on heavy batteries and complex setup of cables [14].

Antenna can be easily integrated into any surface for transmitting signals to a database station that provides information about surface deformations [15]. The simple planar nature, low cost, and smooth adaptability of the antenna sensors will ease the detection process. Apart from detecting cracks, the antenna system also plays a significant role in wireless communication. The antenna sensor works by measuring the shift in its resonant frequency and phase as a response to stimuli [16]. This shift resulted from the variation in physical factors such as temperature, humidity, and the material strain which is developed in the material [17]. Several types of antenna sensors have been developed to date, including temperature and humidity sensors [18], strain sensors [19], adulteration sensors [20], and corrosion sensors [21,22]. The authors Xianzhi Li et al. [23] studied the performance of a feasible patch antenna sensor using temperature and crack detection experiments. A 4 GHz operating wireless sensor is devised by Nan-Wei Chen et al. [24] to monitor crack widening up to 2 mm. In this method, an open-circuited stub is loaded to the antenna for its use as a sensing element. A flexible antenna sensor based on graphene film is proposed by Cong Tong et al. for crack identification and estimation of direction and length in metal structures [25]. A surface crack spread over a large area, along with its length, width, and depth, which are detected using a dual-band microstrip antenna sensor as addressed by Quanhao Pang et al. [26]. The researchers Dan Li et al. [27] proposed a patch antenna sensor that provides invariant results under temperature fluctuations to estimate strain and expansion of crack. The authors Jiaqi Zhang et al. [28] present a dipole antenna sensor designed for strain- and humidity-sensing functions. In a paper by Menon et al. [29], a square open-loop resonator sensor with sensitivities of 36 MHz/mm and 39 MHz/mm is deployed for the early detection of cracks occurring in the ground plane. Mohamed A. Abou-Khousa et al. [30] review a nondestructive testing and evaluation (NDT&E) method to characterize hidden and exposed cracks.

In the present study, a monopole antenna is used as the sensor for crack detection. To the best knowledge of the authors, with the support of ample literature, studies on crack sensing utilizing monopole antenna are limited. The paper is organized as follows: the proposed methodology is discussed in Section 2. Antenna characterizations and responses of the study are addressed under Section 3. Section 4 gives an insight into crack propagation and its effect on antenna resonance. This is followed by the conclusion of the work.

## 2. Methodology

**Figure 1:**
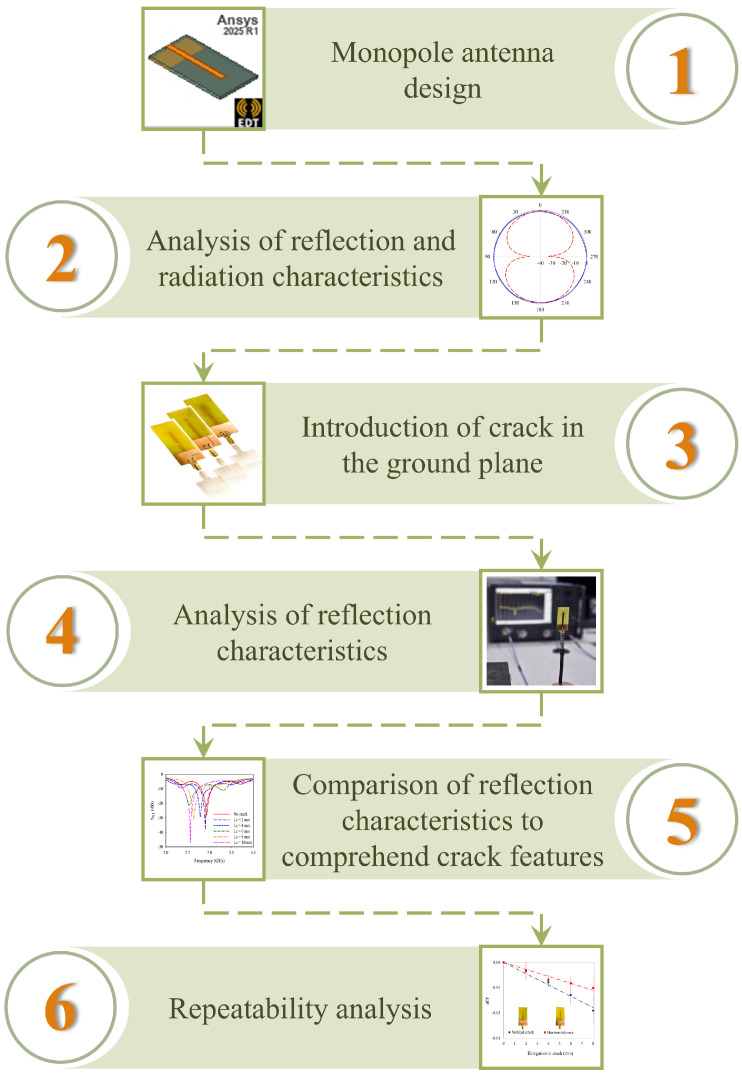
Methodology of the present study.

The methodology for the design and implementation of a monopole antenna sensor is illustrated in Figure 1. A simple monopole antenna is designed to operate at 2.54 GHz on FR4 epoxy as the initial step. The geometrical parameters are calculated from fundamental principles and numerically optimized using the Finite Element (FE) method using Ansys HFSS™ 2025 R1 version. The reflection characteristics of this antenna provide benchmarking for sensor implementation in the later stages. Linear cracks of varied dimensions are incorporated at different locations in the ground plane of the monopole antenna. The simulated responses of the sensor are validated with repeated experiments.

## 3. Antenna Design

This section highlights the analytical design, numerical simulations, and experimental validation of the proposed antenna design.

### 3.1. Analytical Design

**Figure 2:**
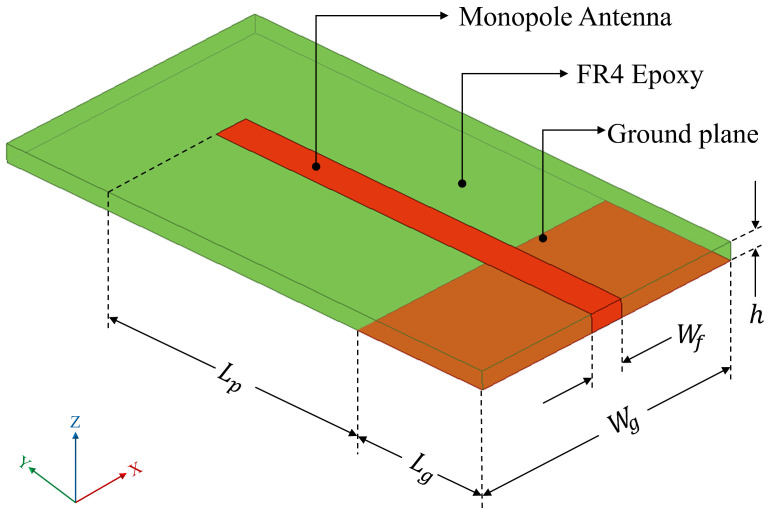
Geometry of the monopole antenna.

A 50 Ω microstrip line is designed to energize the monopole, which has a length of *L*_p_ as depicted in Figure 2. The ground plane dimensions are taken as *L*_g_ × *W*_g_. The design of the monopole antenna follows Equations (1)–(5).

(1) $$L_{p}=W_{g}=\frac{\lambda_{g}}{4}$$

(2) $$L_{g}=\frac{L_{P}}{2}$$

(3) $$\varepsilon_{eff}=\frac{\varepsilon_{r}+1}{2}+\frac{\varepsilon_{r}-1}{2}\sqrt{1+\frac{12h}{W_{f}\;}}$$

(4) $$Z_{0}=\frac{120\pi }{\sqrt{\varepsilon_{eff}}\left[\frac{W_{f}}{h}+1.393+\frac{2}{3}\ln\left(\frac{W_{f}}{h}+1.444\right)\right]},$$

(5) $$\lambda_{g}=\frac{\lambda }{\sqrt{\varepsilon_{eff}}}$$

*λ*_g_ represents the guided wavelength and λ is the operating wavelength in free space. The design is carried out for a characteristic impedance (*Z*_0_) of 50 Ω, which provides the width (*W_f_*) for the microstrip line to energize the monopole antenna. The effective dielectric constant (*ε*_eff_) helps with realizing the monopole antenna on an FR4 epoxy substrate having a relative dielectric constant (*ε*_r_) of 4.4 and height (*h*) of 1.6 mm for the desired operating frequency of 2.54 GHz.

### 3.2. Numerical Simulation Using Finite Element Analysis (FEA)

The geometrical model of the antenna is shown in Figure 2. The dimensional parameters of the monopole antenna are calculated using standard design equations [31]. Subsequently, Finite Element Analysis (FEA) is used to ascertain the physical dimensions to match the design frequency. The simulations were performed in a commercial FEA software—Ansys HFSS^TM^ 2025 R1 version. To ensure finite discretization, the mesh size is approximated to ≤λ/20 [32]. The optimized geometrical parameters are given in Table 1, and the properties of the selected substrate material (FR4 epoxy) are given in Table 2.

**Figure 3:**
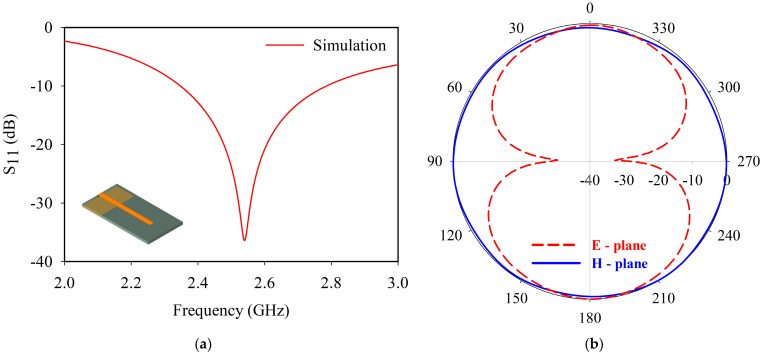
Monopole antenna: (**a**) return loss; (**b**) radiation pattern.

Figure 3a illustrates the simulated return loss of the monopole antenna. The antenna provides good impedance matching with a reflection coefficient of 0.0112 (−39 dB) at 2.54 GHz. A 2:1 VSWR bandwidth of 444 MHz is observed for the monopole antenna. Figure 3b represents the radiation pattern of the monopole antenna, and the obtained pattern is in line with the classical omnidirectional monopole behavior. The antenna acquires a gain of 2.70 dBi at the resonance frequency.

The characteristics of the antenna affirm that the reflection properties of the monopole antenna can be taken as the governing parameter for sensor analysis.

## 4. Analysis of Monopole Antenna as Sensor

The antenna’s ability to detect cracks is analyzed by introducing a slot at the edge of the ground plane. The crack length (*L*_c_), width (*W*_c_), and position (d) are varied to understand the influence of these parameters on the resonance behavior of the antenna. This analysis is carried out in the longitudinal and transverse (Y and X) directions, ensuring that the whole ground plane surface is covered.

### 4.1. Variation in Crack Propagation in Longitudinal (Y) Direction

**Figure 4:**
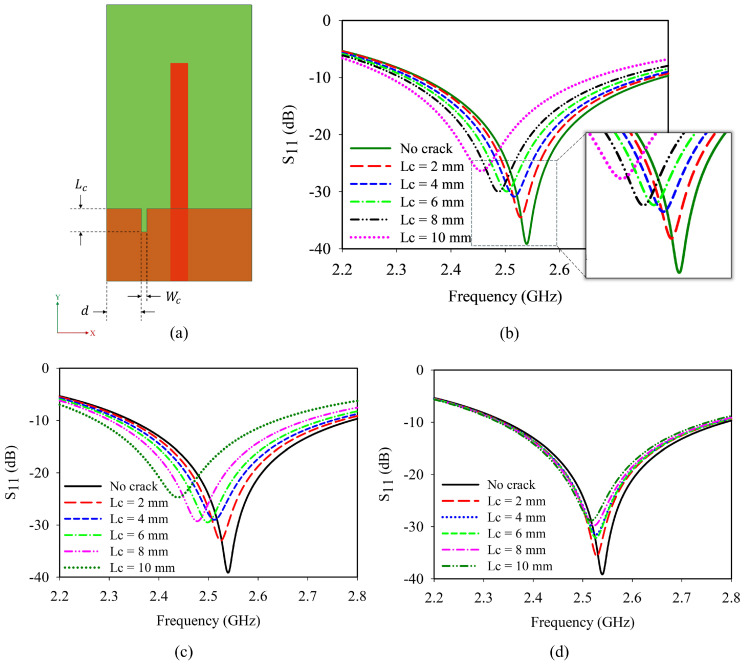

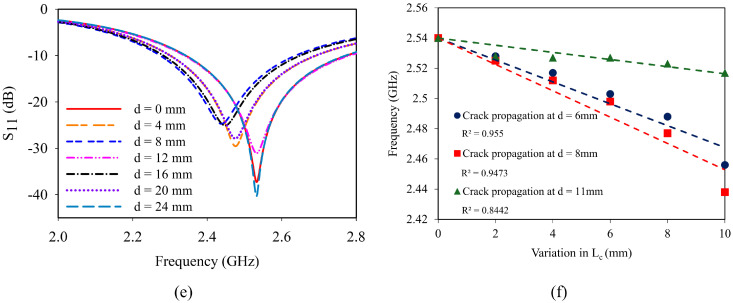
Crack propagation in Y-axis: (**a**) geometry of the antenna sensor; reflection coefficient of crack propagation in Y-axis: (**b**) elongation of crack at d = 6 mm; (**c**) elongation of crack at d = 8 mm; (**d**) elongation of crack at d = 11 mm; (**e**) dependence of distance variation for a 10 mm-long crack; (**f**) parametric analysis of crack length and frequency.

In the initial analysis, a crack having length (*L*_c_) and width (*W*_c_) is introduced at a distance (d) from the edge of the ground plane as shown in Figure 4a. The variation in crack along the Y direction is analyzed in two different scenarios.

Initially, the length of the crack (*L*_c_) is increased from 2 mm to a maximum of 10 mm at three different locations (d) 6 mm, 8 mm, and 11 mm from the edge of the ground plane. Figure 4b shows the frequency response of the antenna when the crack is introduced at 6 mm. The resonance frequency is found to shift towards the lower side of the spectrum in this case. Similar trends are observed when the crack length is varied at a distance of 8 mm and 11 mm, which is depicted in Figure 4c,d.

Secondly, a 10 mm crack is introduced and traversed from d = 0 mm to d = 24 mm. With respect to the monopole, on either side, there is a similar variation due to the symmetry of the sensor as shown in Figure 4e. The crack propagation shows a linear relation with frequency as depicted in Figure 4f. The analysis shows that the resonance shifts are predominantly visible during crack propagation. Moreover, a minute crack initiation can also be detected from the frequency responses.

### 4.2. Variation in Crack Propagation in Transverse (X) Direction

**Figure 5:**
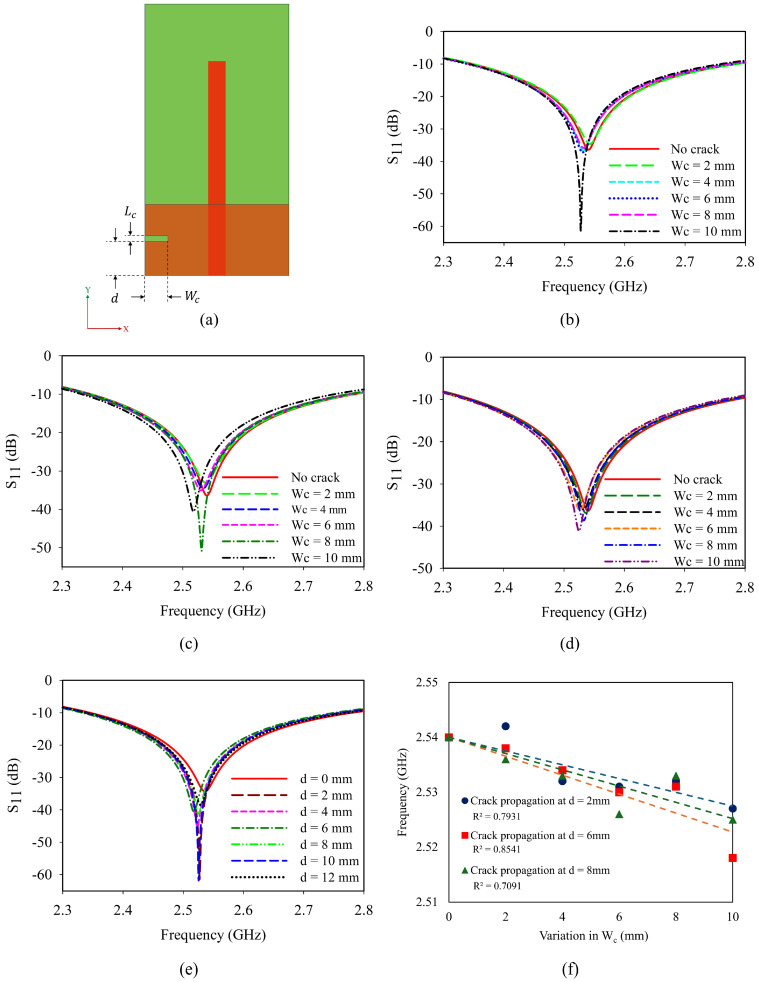
Crack propagation in X-axis (**a**) geometry of the antenna sensor; reflection coefficient of crack propagation in X-axis: (**b**) elongation of crack at d = 2 mm; (**c**) elongation of crack at d = 6 mm; (**d**) elongation of crack at d = 8 mm; (**e**) dependence of distance variation for a 10 mm long crack; (**f**) parametric analysis of crack width and frequency.

A similar analysis is carried out for the propagation of a crack along the X-axis. Figure 5a illustrates the variation in resonance while positioning the crack along the X-axis. Analysis is carried out to find the influence of crack width (*W*_c_) and position (d) in the reflection characteristics of the sensor. The observed results are presented in Figure 5b–f.

As the crack propagates in the ground plane, the fringing introduces an effective dielectric constant, *ε*′_eff_, instead of *ε*_r_, which will depend on the length and width of the crack. This effective dielectric constant depends on the effective area (*A*_eff_) of the crack, which is accommodated by the variations in the crack dimensions. Similarly, to accommodate the variations in the *ε*′_eff_ in the analytical model, a geometrical correction factor (*γ*) is introduced. The value of *γ* is obtained by combined analytical and numerical simulations. *d*_eff_ is the dimension of the crack represented as *L*_c_ or *W*_c_ in the ground plane. If the crack is expanding longitudinally in the X-axis, the *d*_eff_ will be equal to the respective *L*_c_ value, and if *d*_eff_ is propagating in the Y-axis, it will be equal to *W*_c_.

(6) $$\varepsilon_{\mathrm{eff}}^{\prime }\;≅\;\varepsilon_{r\;}-\;x\;\left(\varepsilon_{r}\;-\;1\right)$$

where,

(7) $$x\;≅\;\gamma \;\frac{A_{\mathrm{eff}}}{d_{\mathrm{eff}}^{2}},$$

*γ* is the geometrical correction factor, *γ* = 1.25 for *ε_r_* ≤ 6.2,

(8) $$d_{\mathrm{eff}}=\left\{\begin{array}{c} L_{c},\;for\;horizontal\;crack\\ W_{c},\;for\;vertical\;crack \end{array}\right.$$

**Figure 6:**
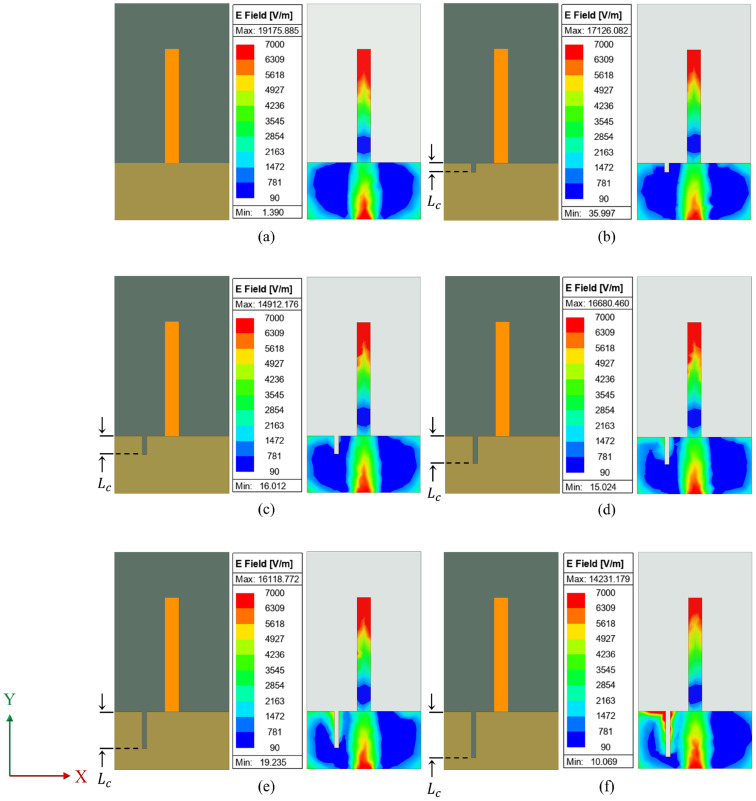
Electric field variation with change in crack length (*L*_c_): (**a**) *L*_c_ = 0 mm; (**b**) *L*_c_ = 2 mm; (**c**) *L*_c_ = 4 mm; (**d**) *L*_c_ = 6 mm; (**e**) *L*_c_ = 8 mm; (**f**) *L*_c_ = 10 mm.

**Figure 7:**
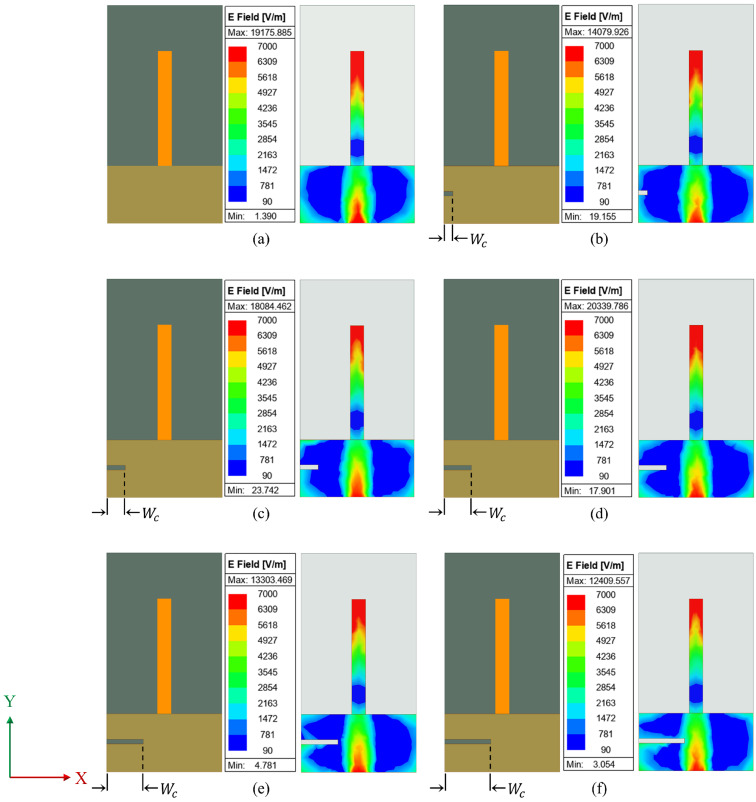
Electric field variation with change in crack width (*W*_c_): (**a**) *W*_c_ = 0 mm; (**b**) *W*_c_ = 2 mm; (**c**) *W*_c_ = 4 mm; (**d**) *W*_c_ = 6 mm; (**e**) *W*_c_ = 8 mm; (**f**) *W*_c_ = 10 mm.

In effect, the crack dimension perturbs the surface current through the ground plane, which leads to variation in effective capacitance, thus affecting the resonance characteristics. This is clearly depicted in the current distribution in Figure 6 and Figure 7.

A crack is generally a discontinuity in the metallic ground, which redistributes the electric fields around it. When the crack is closer to the monopole, the electric field gets affected at a higher rate due to higher fringing. Figure 6 illustrates the field variation when the length of crack in ground plane (*L*_c_) varies from 2 mm to 10 mm along a longitudinal direction at 6 mm from the edge. As the length of the crack increases, the electric field gets intensified as shown in Figure 6f. Similarly, the variation in the electric field while the crack propagates along the X-axis is illustrated in Figure 7. The width of the crack in the ground plane (*W*_c_) varies from 2 mm to 10 mm along the X-axis. As stated above, the electric field gets affected by the crack propagation and is clearly visible in Figure 7f. When the crack is propagating along Y-axis, a significant re-distribution of the electric field occurs closer to the monopole. Figure 6 shows that as the length of the crack expands, so does the distribution of the electric field surrounding it, owing to increased fringing. However, for a crack oriented along the X-axis, there is lesser effect on electric field distribution as shown in Figure 7. As a result, the sensor’s sensitivity is found to be higher in crack propagation along the Y-axis than in the X-axis.

**Figure 8:**
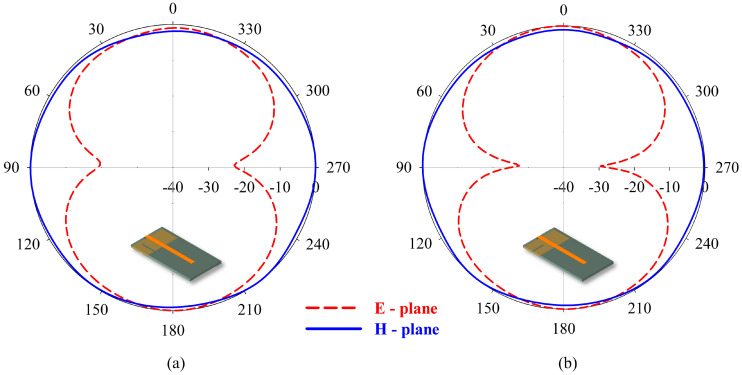
Radiation pattern of crack having a length of 10 mm positioned at d = 6 mm along: (**a**) Y-axis; (**b**) X-axis.

The initiation or propagation of a crack has a minor effect on the gain of the sensor. When the position of crack is in phase with the current distribution in the ground plane, the field undergoes constructive interference, leading to better sensitivity and radiation patterns, which can be seen in Figure 8a,b. The effect of the crack in the bandwidth and gain in all the test case scenarios are presented in Table 3. Bandwidth is found to be less influenced by the introduction of crack. A consistent gain is observed in the case of crack propagation at different positions, making the antenna feasible for wireless sensing. The sensor can detect the crack propagation in both X and Y directions as observed in FEA.

On the inclusion of a crack in the ground plane, the variation in gain is minimal. However, we have observed a variation of 16% in bandwidth in Y direction crack propagation. This is due to the asymmetry that the crack in the Y direction creates in the ground plane, which is more predominant than in the X direction. Now, this study is extended experimentally, and the observations are presented in next section.

## 5. Experimental Assessment

**Figure 9:**
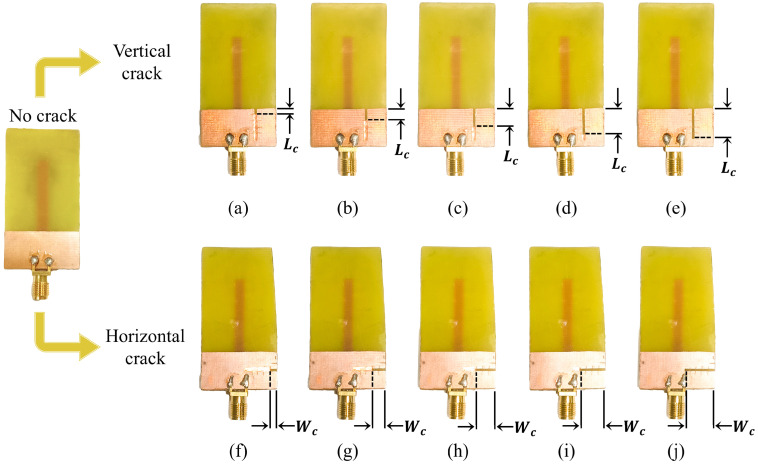
Crack propagation along the vertical and horizontal axis: (**a**) *L*_c_ = 2 mm; (**b**) *L*_c_ = 4 mm; (**c**) *L*_c_ = 6 mm; (**d**) *L*_c_ = 8 mm; (**e**) *L*_c_ = 10 mm; (**f**) *W*_c_ = 2 mm; (**g**) *W*_c_ = 4 mm; (**h**) *W*_c_ = 6 mm; (**i**) *W*_c_ = 8 mm; (**j**) *W*_c_ = 10 mm.

**Figure 10:**
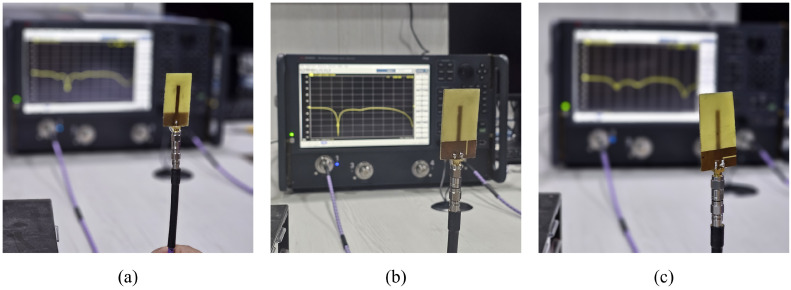
Experimental setup of antenna sensor: (**a**) no crack; (**b**) crack in Y-direction; (**c**) crack in X-direction.

**Figure 11:**
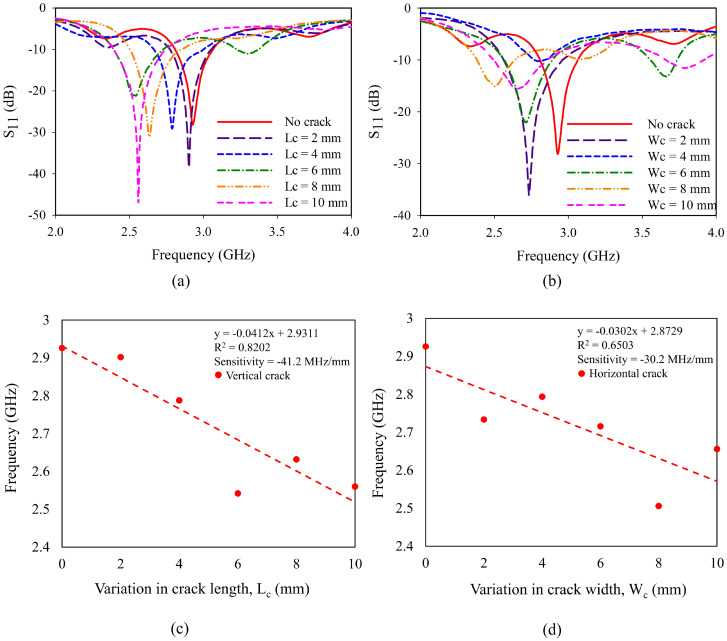
Variation in reflection coefficient with elongation of crack: (**a**) crack in Y-direction; (**b**) crack in X-direction; variation in frequency with elongation of crack: (**c**) crack in Y-direction; (**d**) crack in X-direction.

The prototype of monopole sensors is fabricated and is presented in Figure 9. For the characterization, the reflection coefficient of prototypes is measured using Keysight N5227B PNA microwave network analyzer, available at Amrita Vishwa Vidyapeetham. The experimental setup used for the characterization is shown in Figure 10. The variation in the resonant frequency of the monopole due to crack propagation is presented in Figure 11. It is observed from Figure 11a,b, that the resonant frequency varies linearly when the crack propagates along X and Y directions (*W*_c_ and *L*_c_).

**Figure 12:**
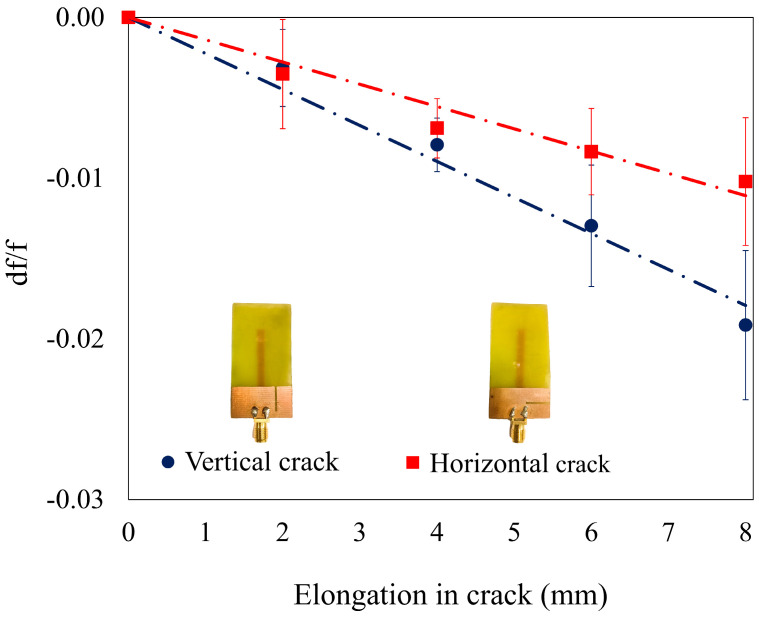
Variation in frequency for repeated measurements.

The sensitivities of the sensor along the Y and X directions are depicted in Figure 11c,d. A higher sensitivity of −41.2 MHz/mm is obtained for the cracks propagating along the Y direction than the other. This higher sensitivity (+36.42%) is due to the higher rate of perturbation of electric field across the ground plane in the vicinity of the monopole. The fabricated monopole antennas were tested five times, and similar results were obtained with minimal standard deviation. The sensor with a crack oriented in the X-axis has a sensitivity of −30.2 MHz/mm. The graph showing repeatability is shown in Figure 12, and the observed variations are linear. A one-way ANOVA analysis is carried out using Tukey’s HSD test for the data obtained during repeatability testing. The corresponding *p*-values for crack propagation in Y-axis and X-axis are found to be 0.9746 and 0.1267. This is higher than the threshold value of 0.05, thus ensuring that the sensors obtain similar results on repeated trials. Table 4 provides a comparison of change in frequency (in %) between the FEA data and experimentally obtained data.

A comparison of the proposed sensor with those available in the literature is studied to analyze the efficacy of the sensor. In comparison with the recently published works, monopole antennas are used for crack detection for the first time. The proposed sensor has good sensitivity in both directions and is comparable with the other configurations seen in the literature, as presented in Table 5.

## 6. Conclusions

This paper explores the possibility of utilizing a monopole antenna as a crack sensor. An antenna operating at 2.54 GHz resonant frequency is designed in the initial stages of the study. The efficacy of the sensor is validated by studying resonant frequency shifts as crack propagates through the ground plane of the antenna sensor. Two individual directional cracks are studied, namely the vertical crack and the horizontal crack spanning along the length and width of the ground plane.

The results obtained from multiple experiments show a linear variation between frequency and crack propagation. A sensitivity of −41.2 MHz/mm and −30.2 MHz/mm is observed for respective sensors having crack propagation along the length and width of ground plane. The field and radiation characteristics are also analyzed to study the characteristics of the antenna sensors. The gain and bandwidth remain nearly constant throughout the study. The results from repeated testing of sensors affirm that the sensor is highly accurate and precise in real-time detection and the monitoring of cracks on the ground plane.

The fabrication process, along with the material properties of FR4 (*ε*_r_ and tan *δ*), significantly affects the sensitivity of the sensor. During the simulation, the metallic parts are assigned perfect E conditions; however, in real-time measurements, instead of perfect E conditions, copper is used, which in turn affects the real and imaginary parts of the impedance. As a result, the accumulative sensitivity of the sensor for sensing the crack is higher in comparison with the simulated result. The wireless-sensing possibilities of the proposed sensor are yet to be explored. Furthermore, the possibility of detecting multiple cracks is another domain of research.

## Figures and Tables

**Table 1:** Optimized parameters of monopole antenna.

Parameters	Optimized Values (mm)
Length of the monopole, *L*_p_	25
Length of the ground, *L*_g_	12.5
Width of the ground, *W*_g_	25
Width of the feedline, *W*_f_	3
Height of the substrate, *h*	1.6

**Table 2:** Properties of FR4 epoxy substrate.

Properties	Values
Dielectric constant, (*ε*_r_)	4.4
Loss tangent (*tan δ*)	0.02
Modulus of Elasticity, E (GPa)	20.4
Poisson’s ratio (*ν*)	0.12
Height of the substrate, *h* (mm)	1.6

**Table 3:** Analysis of crack propagation in gain and bandwidth.

Axis	Presence of Crack	Gain (dBi)	Bandwidth (MHz)
X-axis	No	2.7	444
	Yes	2.83	418
Y-axis	No	2.7	444
	Yes	2.73	378

**Table 4:** Comparison of simulated and fabricated antenna sensors.

Sl. No.	Orientation of Crack	Lc/Wc (mm)	% Change in Frequency	
			Simulation	Measurement
1	No crack	-	0	0
2	Y-axis	2	0.47	0.82
		4	0.90	4.71
		6	1.45	13.12
		8	3.62	10.05
		10	3.26	12.5
3	X-axis	2	0.078	6.56
		4	0.24	4.51
		6	0.39	7.17
		8	0.39	14.35
		10	0.86	9.22

**Table 5:** Comparison of the published crack sensors.

Type of Sensor	Methodology	Substrate (ε_r_)	Frequency (GHz)	Dimension (mm × mm × mm)	Sensitivity
[23]	Patch antenna	RT/duroid 5880 (2.2)	1.95 GHz	-	45.3 MHz/mm
[24]	Open stub in patch antenna	FR4 (4.4)	4 GHz	-	-
[25]	Flexible antenna	Polyethylene terephthalate (3.5)	2.25 GHz	60 × 50 × 0.5	36.82 MHz/mm
			3.1 GHz		
[26]	Microstrip antenna	Rogers 4003c (3.38)	2.8233 GHz	90 × 35 × 1.524	7 MHz/mm
			3.4788 GHz		4.7 MHz/mm
[27]	Patch antenna	RT/duroid 6202 (2.90)	900 MHz	62 × 119.1 × 0.762	-
[29]	Open-loop resonator	FR4 (4.4)	2.5 GHz	19.8 × 19.2 × 1.6	36–39 MHz/mm
[33]	Cylindrical dielectric resonator	Ceramic (90)	1.318 GHz	24.0 (diameter) × 9.0 (height)	25.4 MHz/mm
[34]	Patch antenna sensor	FR4 (4.4)	1.8 GHz	64 × 44 × 0.6	-
			2.5 GHz		
[14]	Circular patch RFID antenna sensor	Rogers RO4350b (3.66)	902–928 MHz	50.0 (diameter) × 1.524	1.08 MHz/mm^2^
[35]	Microstrip RFID tag antenna	FR4 (4.4)	925 MHz	80 × 84 × 1.5	-
[36]	Circular microstrip patch antenna	Taconic CER-10-0500 (10)	4.70 GHz	35 × 15 × 1.27	−13.43 MHz/0.1 mm
					6.67 MHz/0.1 mm
Present work	Monopole antenna	FR4 (4.4)	2.5 GHz	47.5 × 25 × 1.6	−41.2 MHz/mm −30.2 MHz/mm

## Data Availability

Data available on request.
